# Supplementary material for: Deepbinner: Demultiplexing barcoded Oxford Nanopore reads with deep convolutional neural networks
Source: PLoS Comput Biol. 2018 Nov 20;14(11):e1006583. doi: 10.1371/journal.pcbi.1006583 (PMC6245502; doi:10.1371/journal.pcbi.1006583)
Supplement: S1 Fig — Multiple types of signals were included in the training set to explicitly teach the neural network what a barcode-free signal looks like. The signal amplitude has been normalised to a mean of 0 and a variance of 1. (PDF) [file pcbi.1006583.s005.pdf]

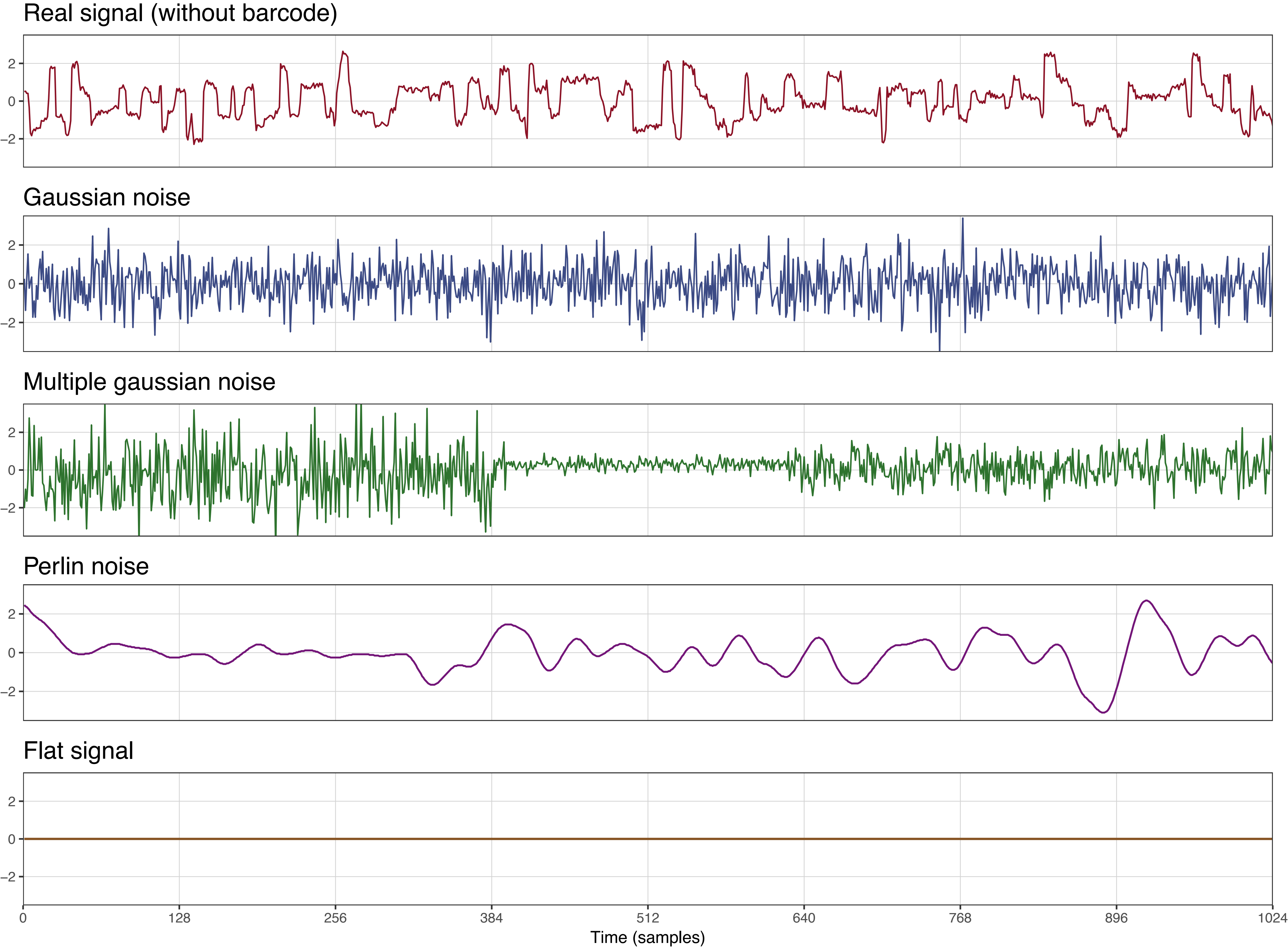
Real signal (without barcode)
Gaussian noise
Multiple gaussian noise
Perlin noise
Flat signal
2
0
−2
0
128
256
384
512
640
768
896
1024
Time (samples)
