# Supplementary figures and images for: Deepbinner: Demultiplexing barcoded Oxford Nanopore reads with deep convolutional neural networks
Source: PLoS Comput Biol. 2018 Nov 20;14(11):e1006583. doi: 10.1371/journal.pcbi.1006583 (PMC6245502; doi:10.1371/journal.pcbi.1006583)

Original signal

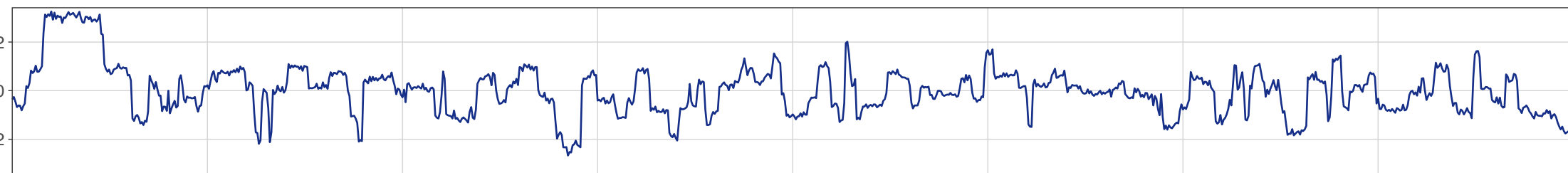

Augmented signals

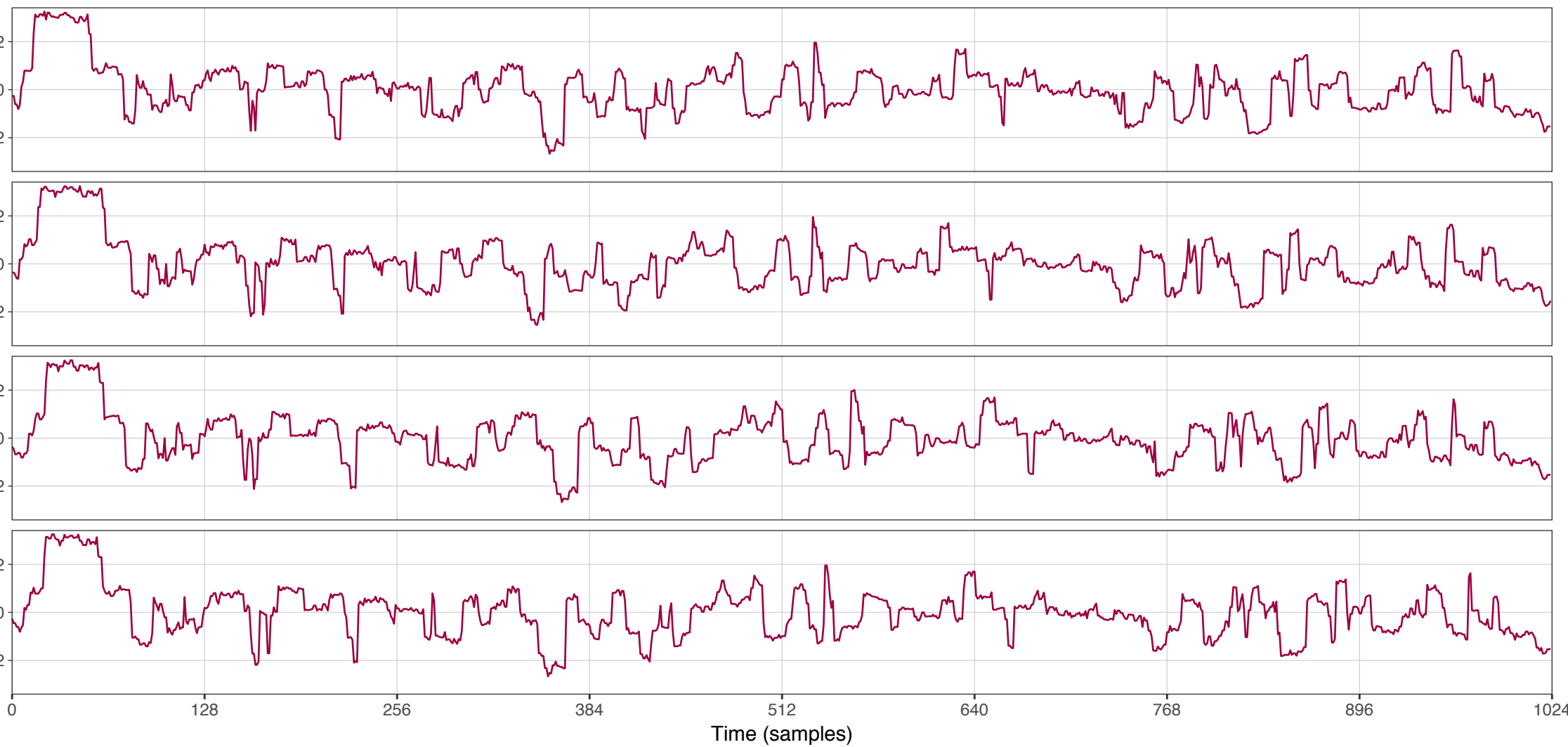

Supplement: S2 Fig — One real training sample can yield multiple additional training samples by distorting the signal along the temporal axis. The signal amplitude has been normalised to a mean of 0 and a variance of 1. (PDF) [file pcbi.1006583.s006.pdf]

Read starts

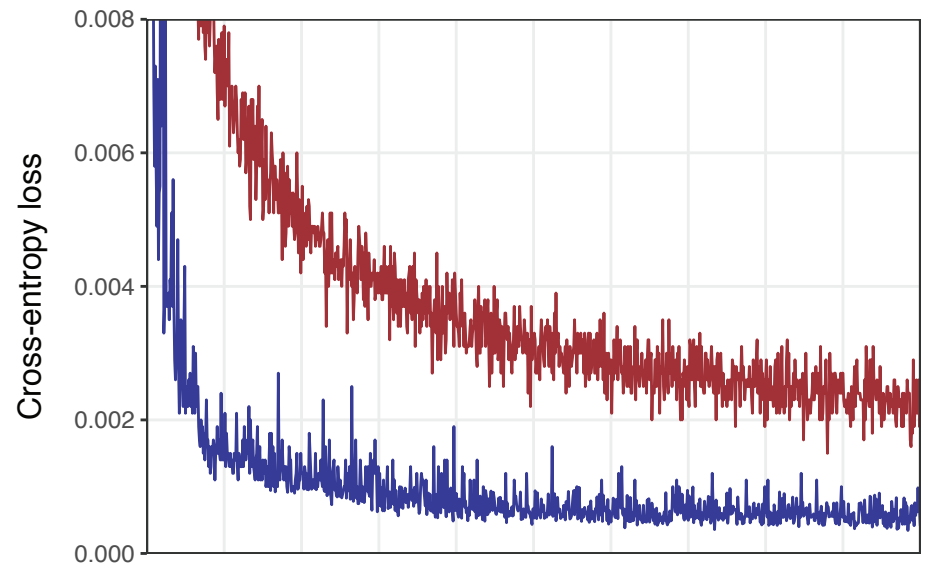

Read ends

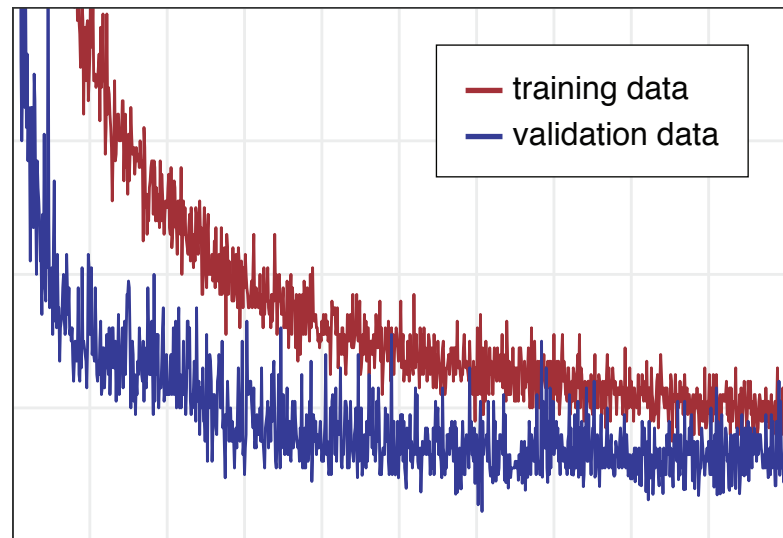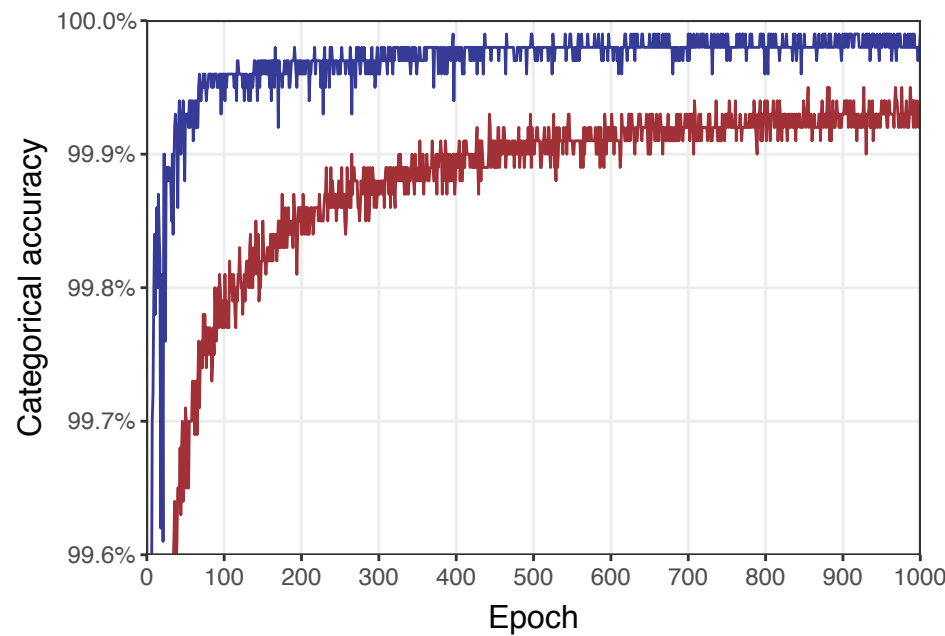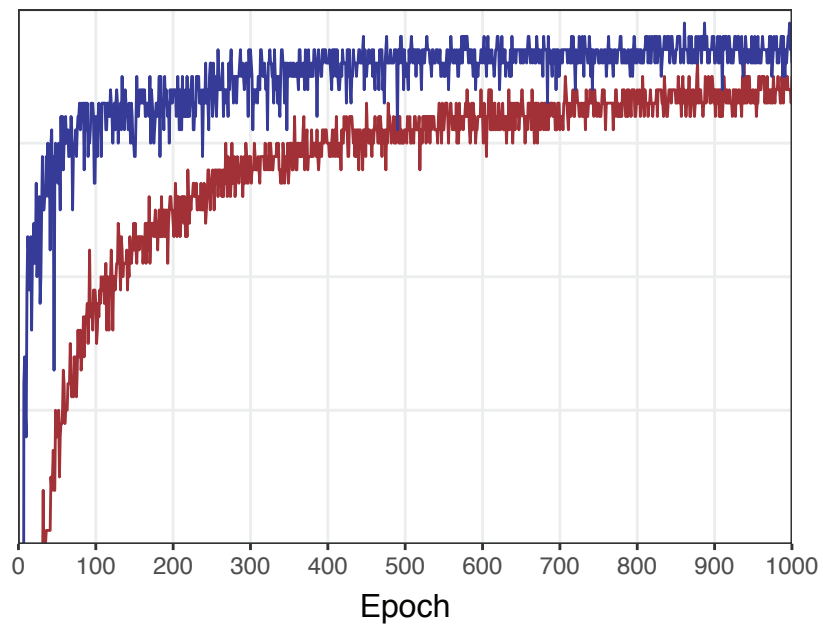

Supplement: S3 Fig — Generated using a random 95:5 training:validation split. Training data has poorer performance than validation data due to data augmentation and training-only layers (Gaussian noise and dropout) in the network. (PDF) [file pcbi.1006583.s007.pdf]

# Precision and recall vs q score

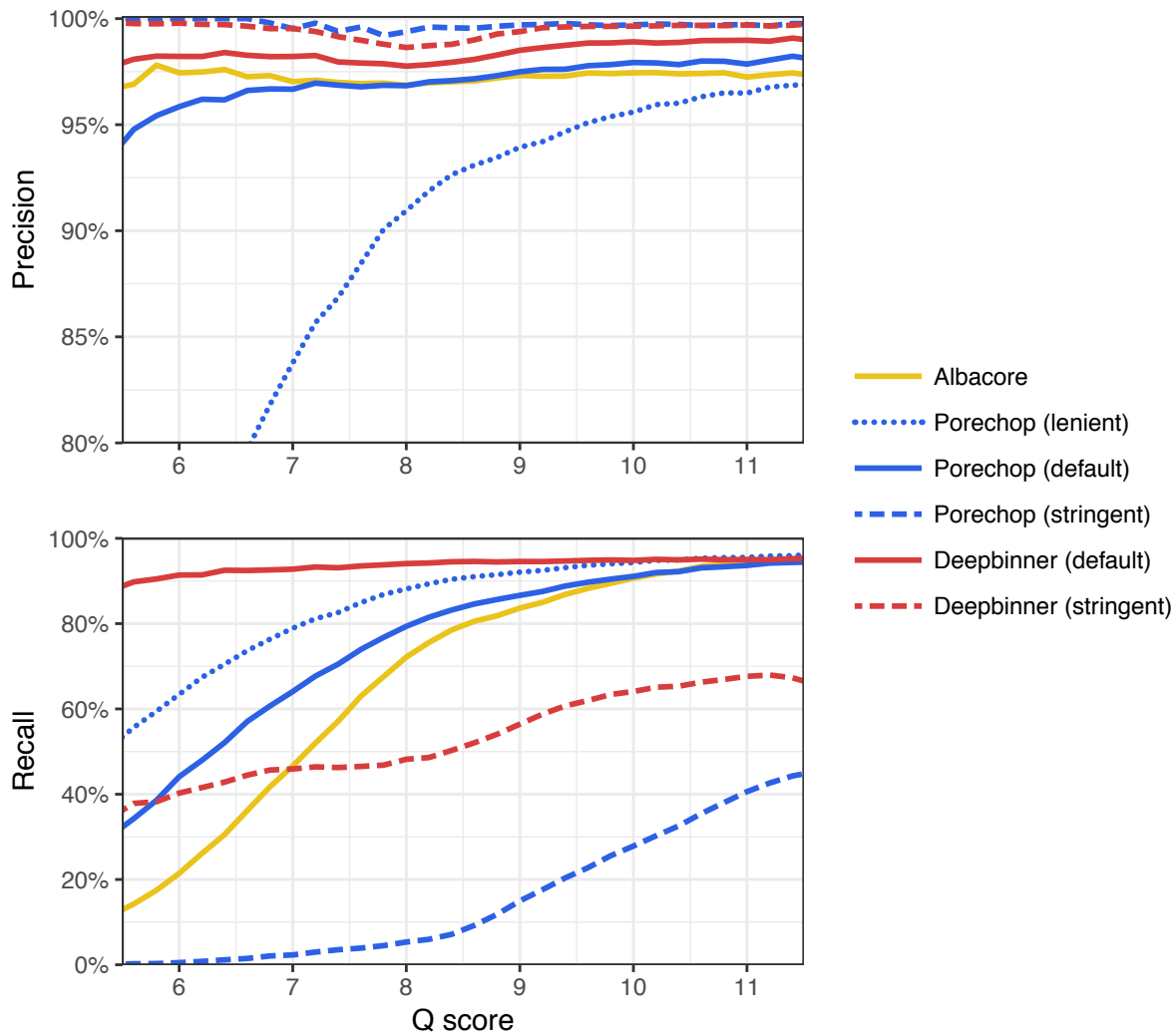

Supplement: S4 Fig — Precision and recall for each tool’s demultiplexing of the amplicon read set as a function of read q score. (PDF) [file pcbi.1006583.s008.pdf]
